# Does China’s national volume-based drug procurement policy promote or hinder pharmaceutical innovation?

**DOI:** 10.3389/fphar.2024.1392239

**Published:** 2024-06-27

**Authors:** Xiujuan Li, Jiachun Xu

**Affiliations:** ^1^ Economics and Management School, Wuhan University, Wuhan, China; ^2^ School of Economics, Jinan University, Guangzhou, China

**Keywords:** China’s national volume-based drug procurement policy, innovation input, innovation output quantity, innovation output quality, multi-period DID

## Abstract

**Introduction:**

The national volume-based drug procurement policy initiated in China since 2018 represents a significant reform in China’s pharmaceutical distribution system. It has largely squeezed out the price bubble of low-end generic drugs, making competition in the pharmaceutical sales segment more intense and transparent. This policy intervenes in the distribution link of the pharmaceutical industry by intensifying market competition, thereby enhancing the innovation willingness and R&D capabilities of pharmaceutical companies.

**Methods:**

Taking the national volume-based drug procurement policy as the policy shock, we used the multi-period difference-in-difference method to study the impact of the policy on innovation input, innovation output quantity and innovation output quality of listed pharmaceutical companies and its impact mechanism.

**Results:**

We found that the volume-based policy can significantly promote the pharmaceutical companies’ innovation input and the innovation output quality, but significantly reduced the innovation output quantity. For innovative and generic drug companies, this policy has limited impact on innovative drug companies, but force generic drug companies to pay more attention to cost control and market positioning, and the quality and cost-effectiveness of R&D output to ensure competitiveness in the market. For bid-winning and non-winning companies, the policy has a greater innovation incentive for non-winning companies than winning companies, by imposing greater survival pressure on non-winning companies, forcing them to increase R&D investment intensity and adopt the innovation strategy of preferring quality to quantity.

**Discussion:**

The results show that the national volume-based drug procurement policy should be expanded to lower drug prices and lighten the medical burden on patients, with enhanced quality and safety supervision. Additionally, it suggests cautious application of such policies to innovative and high-end generic drugs to encourage continued pharmaceutical innovation and industry advancement.

## 1 Introduction

China is the second largest pharmaceutical consumer market in the world, but its drug market has significant differences compared to developed countries such as Europe, the United States, and Japan. In developed countries, generic drugs are used extensively due to their cost-effectiveness, accounting for 80%–90% of the usage share, but only 10%–20% of the drug cost share. In contrast, innovative patented drugs, although used less frequently, make up only 10%–20% of the usage share, yet they occupy 80%–90% of the drug cost share. This market structure not only meets the basic medication needs of the people but also greatly stimulates the innovation and research and development of pharmaceutical companies. However, as a major producer and user of generic drugs, China has a high usage share of generic drugs at 80%, but unlike developed countries, the cost share of generic drugs is also high at 80%. The usage and cost share of innovative patented drugs are significantly lower, with usage less than 5% and cost share less than 10%. The vast majority of medical insurance drug costs are spent on generic drugs or even unevaluated drugs, which neither allows patients to access high-quality drugs and the latest good drugs, nor provides sufficient financial support for pharmaceutical companies to innovate.

To tackle the problem of inflated prices and a disproportionately large share of costs for generic drugs, which crowd out pharmaceutical innovation, China is leveraging its institutional strengths and adheres to market principles by organizing national volume-based procurement of generic drugs through National Healthcare Security Administration. The national volume-based procurement policy, officially launched in 2018, aims to select generic drugs that are of consistent quality with the branded drugs from those that have passed the generic drug quality consistency evaluation, and to reduce their prices. This initiative also seeks to decrease corporate transaction costs, control medical insurance expenditures, guide hospitals towards standardized drug usage, and thus refine China’s volume-based drug procurement mechanisms and market-oriented drug pricing mechanisms. Since the initial implementation of the “4 + 7″pilot city volume-based procurement in 2018, by the end of 2022, China has conducted seven batches and eight rounds of procurement, covering a total of 294 types of generic drugs. Based on the results of the previous seven batches of procurement, the policy has significantly reduced the drug procurement prices for medical institutions, with an average price reduction of about 50%. About 60% of the savings from the national volume-based procurement of generic drugs have been allocated to national negotiations for innovative drugs. This has achieved “vacating cage to change bird” of medical insurance funds between generic drugs and innovative drugs, as well as a similar strategic shift from sales expenses to research and development costs within pharmaceutical companies.

Prior researches have indicated that the implementation of volume-based procurement policy has the potential to effectively control drug prices, enhance the efficiency of drug distribution, and improve drug quality. This approach is widely adopted in the global public drug procurement, as evidenced by studies conducted by (Weinstein, 2006; Hu and Schwarz, 2011; Zhou et al., 2017; Bruhn et al., 2018). In contrast to the GPO model implemented in other countries, China’s national volume-based drug procurement involves a government-led direct procurement mechanism. As buyers of drugs and medical devices, the government uses its market advantages and economies of scale through volume-based procurement to sign contracts with pharmaceutical companies, lock in a certain quantity with lower prices. This approach aims to achieve cost-effective procurement and ensure the acquisition of high-quality drugs with lower price.

Volume-based procurement at the government level can be more efficient than centralized procurement by medical institutions, and can effectively reduce drug prices (Vogler et al., 2017). Lu et al. (2021) showed that the implementation of volume-based procurement policy can provide substantial reductions in medicine prices within a market-oriented competitive environment. Lin et al. (2022) proposed that the implementation of volume-based procurement policy has been found to be a beneficial strategy in reducing drug costs, medical expenses, and overall costs, hence alleviating the medical burden on patients. In terms of pharmaceutical firms, the volume-based procurement policy has compressed their profit space, impacted their survival and development, and made the pharmaceutical industry face a reshuffle (Zhou et al., 2022). The adoption of the volume-based procurement policy will exert tremendous pressure on pharmaceutical production enterprises, causing them to face survival difficulties (Sun et al., 2022). Tan and Wu (2022) also found that the volume-based policy poses pressure on the production and operation of pharmaceutical firms, but will promote to improve the speed of industrial transformation and upgrading.

The pharmaceutical industry, being a sector with a high concentration of innovation, is inevitably influenced by the indirect effects of volume-based procurement policies on the innovation of pharmaceutical companies. Firstly, the volume-based procurement policy can influence the innovation of pharmaceutical companies by enhancing the countervailing power. The procurement institutions of public hospitals in China have a large scale and stable purchasing needs, which can form an absolute buyer market power. Therefore, when purchasing drugs, they can control market prices to a certain extent, negotiate with suppliers, and reduce prices, thereby enhancing their bargaining power in the market. The greater the buyer market power, the greater the competition among suppliers, which in turn encourages suppliers to adopt differentiated innovation to gain market share (Becker and Peters, 2000; Szajnfarber and Weigel, 2007; Hommen and Rolfstam, 2008). Kohler and Rammer (2012) conducted a study on 1129 companies from the manufacturing and service industries in Germany and found that buyer power has a negative impact on the R&D activities of suppliers. Li et al. (2014) constructed a dynamic game model from the perspective of buyer’s countervailing power to study the impact of downstream retailers on the innovation of upstream companies, and found that if the terminal retailers have buyer’s countervailing power, the enhancement of this power will inhibit the quality innovation of manufacturers, leading to a decrease in equilibrium price and an increase in equilibrium output. Secondly, the volume-based procurement policy can influence the innovation of pharmaceutical companies by enhancing the competitive structure of the industry. The volume-based procurement is a formal and highly competitive procurement method. Huang and Tao (2020) discussed from the perspective of market size and entry barriers that the drug collection policy can enhance industry concentration, thereby achieving the goal of promoting innovation in pharmaceutical companies. Hu et al. (2021) also proposed that the volume-based procurement policy will accelerate the enhancement of corporate competitiveness and provide momentum for innovation.

Integrating the above research, the current academic focus on the study of national volume-based drug procurement policy is mainly concentrated on drug prices and the financial performance of pharmaceutical companies. A small number of studies have discussed the impact of policies on corporate innovation from the perspective of buyer’s countervailing power and text research, but no empirical research has been conducted.

Therefore, this study makes the following two contributions: 1) Previous studies have shown that the national volume-based drug procurement policy can increase the R&D investment of pharmaceutical companies (Ding, 2021; Li and Shen, 2022). This study takes the policy of drugs as a quasi-natural experiment and focuses on the innovation of pharmaceutical companies, systematically studies the impact of the policy on the innovation strategies of pharmaceutical companies from three aspects: innovation input intensity, innovation output quantity, and innovation output quality; 2) From the perspective of the regulations on drug procurement bidding, the impacts of drug procurement policy on innovative and generic drug companies, winner and non-winner, are different. This study takes heterogeneity tests on the impact of volume-based drug procurement policy on the innovation strategies between these companies.

## 2 Theoretical analysis and research hypothesis

### 2.1 Theoretical analysis

Drug is a special kind of commodity with a high value of technological content, and there is a large information gap between the producers and users of drugs. Therefore, the supply side of drugs inherently has a monopolistic nature. Faced with pharmaceutical companies in a monopolistic position, public medical insurance payers can only become an effective demand-side force to counterbalance the supply side by pooling the scattered demands of more and more patients, that is, the buyer’s countervailing power proposed by Galbraith (2017). The national volume-based drug procurement policy is a formal, price-anchored, highly competitive procurement model. Large pharmaceutical companies, due to economies of scale, can partially offset the negative impact brought by the decline in drug prices. However, small and medium-sized pharmaceutical companies are at a disadvantage in cost control and drug production technology, and their economic strength is also difficult to support the winning bid price lower than the cost, so the volume-based procurement policy will have a serious exclusion effect on small and medium-sized pharmaceutical companies with small scale, low specialization, and insufficient production capacity. This will lead to an increase in the concentration of the pharmaceutical industry. This study elaborates on the impact of the national volume-based drug procurement policy on company innovation from the countervailing power theory and market competition structure theory.

#### 2.1.1 Countervailing power and innovation

Traditional industrial organization theory primarily focuses on the strategies of sellers, typically assuming that buyers are passive price takers, accepting all seller prices that are below their demand curve. While this assumption may accurately describe the purchasing decisions of individual consumers in the retail market, in the markets for industrial products, wholesale, and intermediate goods, a few sellers usually compete with each other to win the business of a few large buyers. Galbraith first introduced the concept of countervailing power in 1952, and he believed that strong buyers constitute a positive constraint on the monopolistic power of sellers.

There are three distinct opinions about the impact of countervailing power on corporate innovation. Countervailing power can promote innovation.

Countervailing power promotes innovation. Katz (1987) argued that larger buyers can more reliably threaten backward integration, thereby exerting greater pressure on suppliers to improve quality. Scherer and Ross (1990) proposed that large buyers’ purchase orders are more likely to break potential collusion among suppliers, thereby promoting innovation within the industry. Inderst and Wey (2003) argued that buyer consolidation may prompt suppliers to choose a production technology that potentially improves welfare. Chen (2007) considered the impact of buyer consolidation on product diversity. Buyer’s countervailing power can make the relationship between buyers and sellers more stable, and this good relationship can promote the integration of the company’s supply chain, reduce financial constraints, and make companies more confident in actively investing in projects, such as R&D innovation (Ak and Patatoukas, 2016).

Countervailing power hinders innovation. The higher the buyer’s countervailing power, the greater the bargaining power of major customers, and the higher the company’s costs and financial risks (Albuquerque et al., 2014; Irvine et al., 2016). Because companies have asymmetric dependence on major customers, losing a major customer can constitute a crisis for the company (Gulati and Sytch, 2007). These companies will retain more cash due to this risk, which may reduce R&D investment (Crawford et al., 2020).

There is a U-shaped relationship between countervailing power and innovation. Patatoukas (2012) found that the “inhibitory” and “promotional” effects of buyer concentration on corporate innovation vary with different levels of buyer concentration. When buyer concentration is below a certain level, as buyer concentration increases, the diversity level of buyers decreases, and at this time, the inhibitory effect on innovation is dominant. When buyer concentration is above this level, the interdependence between suppliers and customers tends to stabilize, and joint investment in proprietary assets and information sharing can stimulate the supplier’s innovation ability, and at this time, the promotional effect on innovation is strong. Shen et al. (2018), using a sample of listed companies in Shanghai and Shenzhen from 2007 to 2015, found a nonlinear U-shaped relationship between buyer concentration and corporate innovation.

#### 2.1.2 Market competitive structure

Regarding the impact of market competition structure on corporate innovation, there are three opinions. Competition can hinder innovation. The Schumpeter hypothesis posits that large corporations possess a distinct edge in procuring funding for ventures involving risky innovations. This advantage stems from their ability to allocate a substantial percentage of the required capital from their own resources, as well as their increased likelihood of securing loans due to their heightened liquidity. Moreover, large corporations can share fixed costs in large-scale sales, thereby reducing unit production costs. Hence, it may be argued that innovation tends to yield more profitability inside larger corporations. Large corporations are generated on the assumption that the industry is an incomplete competitive market. Additionally, large corporations can conduct multiple innovation initiatives concurrently, thereby diversifying the risks associated with R&D (Schumpeter, 1976; Kamien and Schwartz, 1982; Cohen and Levin, 1989). Yu et al. (2021) examined the impact of the implementation of the Anti-Monopoly Law as a policy shock, and found that this legislation had a detrimental effect on the market dominance of monopolistic companies, hence hindering company innovation.

Competition can promote innovation. Kamien and Schwartz (1982) conducted a review of empirical literature before the late 1970s and found that the relationship between market structure and innovation activity is contrary to the Schumpeter hypothesis. The presence of technological opportunities that enable cross-industry changes has a significant role in driving innovation activity (Levin et al., 1985; Cohen et al., 1987; Geroski, 1990; Blundell et al., 1995). Yu et al. (2016) found in their research on industrial policies that government-supported industries tend to exhibit a tendency towards easing market entry, fostering rivalry within the sector, and stimulating innovation among enterprises.

There is an inverted U-shaped relationship between competition and corporate innovation. Aghion et al. (2005) conducted a study examined the correlation between competition and innovation, using Schumpeter’s growth framework as a basis, and found a non-linear relationship between competition and innovation, characterized by an initial positive effect of competition on innovation. However, as competition becomes more intense, the incentive for entrepreneurial innovation diminishes. Peroni and Ferreira (2012) used Luxembourg’s structural business statistics to study the empirical relationship between market competition and innovation, and found that the relationship between competition and innovation is nonlinear, with the crucial factor being the efficient utilization of production inputs.

### 2.2 Research hypothesis

The national volume-based drug procurement leverages its own purchasing scale advantage to centrally purchase drugs and medical devices. By centralizing end consumers to form a buyer’s monopoly, it sets up access standards for the consistency evaluation of generic drugs for drug suppliers, and forms a drug pricing mechanism based on market bidding rules, which belongs to the socialization of government procurement. The national volume-based drug procurement policy can enhance the bargaining power of buyers, reduce the prices of medicines, and increase the concentration of the industry, thereby affecting the innovation investment of pharmaceutical companies. Also, the policy reshapes the mechanism of generic drug bidding and the independent pricing of original patented drugs, compresses the profit space of drugs, and intensifies market competition. However, innovative drugs have not been included in the scope of volume-based procurement. Faced with such a market environment, in order to gain greater development space and competitive advantage, companies will inevitably have a stronger motivation to carry out technological innovation. It can be said that volume-based procurement stimulates and enhances the innovation motivation and enthusiasm of enterprises, and thereby promotes enterprises to increase their R&D investment. Based on the analysis, this study proposes the following hypothesis.

Hypothesis 1 The national volume-based drug procurement policy promotes innovation investment in pharmaceutical firms.

The impact of the national volume-based drug procurement policy on the innovation output of pharmaceutical firms remains unknown, with the selection of innovation strategies by firms being highly influenced by the strength of the firms themselves and the surrounding environment. The dynamic adaptation of a company to its environment inevitably creates a connection between outcomes, evolution, and strategy, where the company’s innovation output is closely related to the choice of innovation strategy (Xu et al., 2014). Effective innovation strategies can help improve innovation performance, but incorrect innovation strategies may diminish the positive influence of other suitable mechanisms on innovation performance (Cesário and Fernandes, 2019).

The policy has stimulated the market’s demand for high-quality, cost-effective drugs by reducing drug prices. Such a market environment encourages pharmaceutical companies to enhance the competitiveness of their products through innovation, thereby standing out in fierce market competition. To maintain or strengthen their market position, pharmaceutical companies may increase their R&D efforts, promoting the development of new products and the improvement of existing ones, to provide drugs with greater clinical value. Under the guidance of policy, the government may provide a series of incentives to support innovation, such as R&D subsidies, tax incentives, and expedited approval channels, all of which can effectively promote the innovation output of pharmaceutical companies. However, the volume-based procurement policy may lead the market to favor price competition over innovation competition, causing pharmaceutical companies to reduce their investment in innovative projects due to financial pressure. Instead, they may shift more focus to cost control and price advantages rather than innovative research and development, ultimately suppressing the innovative output of pharmaceutical companies. Based on this, this study proposes the following hypothesis.

Hypothesis 2a The national volume-based drug procurement policy will increase the innovation output of pharmaceutical companies.

Hypothesis 2b The national volume-based drug procurement policy will reduce the innovation output of pharmaceutical companies.

The national volume-based drug procurement policy is primarily aimed at the generic drug market, with the goal of reducing drug prices through market competition, improving the accessibility of drugs, and also promoting the healthy development of the pharmaceutical industry. This policy has a significant impact on both innovative drug companies and generic drug companies. For innovative drug companies, the procurement policy may provide more space for the payment of innovative drugs by saving medical insurance funds, indirectly promoting the research and development activities of innovative drug companies. As the centralized procurement policy reduces the prices of generic drugs through market competition, innovative drug companies may be motivated by both market and policy to increase R&D investment to develop more clinically valuable new drugs, thereby maintaining an advantage in competition.

In contrast, generic drug companies face price pressure under the policy and may need to reallocate limited resources to adapt to market changes. Generic drug companies are forced to transform and increase their investment in R&D for high-quality generic drugs or innovative drugs to maintain market competitiveness. The centralized procurement policy may reduce the quantity of innovation output in the short term because companies need to pay more attention to cost control and market positioning. However, in the long run, generic drug companies will pay more attention to the quality and cost-effectiveness of innovation output, improving product quality through technological innovation and process optimization to ensure competitiveness in the market. Some powerful generic drug companies will transform towards the field of innovative drugs, thereby enhancing the overall quality of innovation output. Based on this, this study proposes the following hypothesis.

Hypothesis 3 Generic drug companies face greater survival pressure than generic drug companies, and will pay more attention to innovation output quality.

The impact of the national centralized drug procurement policy on winning and non-winning companies is different. After the implementation of the volume-based procurement policy, non-winning pharmaceutical companies face greater survival pressures. The main reason is that the procurement policy ensures the market share and sales volume of the winning drugs, while non-winning companies lose this market guarantee. In this market environment, in order to maintain competitiveness and market share, non-winning pharmaceutical companies may place more emphasis on innovation to improve the technical content and clinical value of their products. Increased investment in innovation can help these companies develop new products with higher added value, such as accelerating the research and development of varieties with large market size, few competitors, and high imitation thresholds, thereby standing out in the fiercely competitive market. At the same time, non-winning companies will also pay more attention to the quality of innovative output to ensure that their products can meet market demands in terms of efficacy, safety, and cost-effectiveness, thereby enhancing the market competitiveness of their products. The sharp increase in survival pressure will prompt non-winning companies to adjust their strategic direction, transforming from relying on traditional generic drugs to the research and development of first generics, difficult-to-imitate drugs, and innovative drugs. Based on this, this study proposes the following hypothesis.

Hypothesis 4 Non-winning pharmaceutical companies face greater survival pressure than bid-winning pharmaceutical companies, and will pay greater attention on innovation input and innovation output quality.

## 3 Research design

### 3.1 Data

This study uses 416 listed pharmaceutical companies classified in the pharmaceutical and biological industry in the Shenwan Industry Classification (2014) as the initial sample. After excluding incomplete data and ST (delisting risk warning and other risk warnings implemented by the exchange) companies, a total of 164 pharmaceutical companies entered the final sample for a total of 1312 annual observations. The official implementation time of the national drug volume-based procurement policy is 2018. In order to compare the implementation of the volume-based procurement policy before and after, this study selects the research period from 2015 to 2022. This study manually organized the directory of pharmaceutical listed companies, its subsidiary and second-tier subsidiaries, and then collected volume-based procurement drug production companies based on the list of listed drug directory, confirming the listed pharmaceutical companies included in the scope of volume-based procurement. The data of drugs in the national volume-based drug procurement policy is sourced from the Shanghai sunshine Medical Procurement All-in-One. The financial data at the company level is sourced from CSMAR, and the company’s patent data is sourced from CNRDS.

### 3.2 Model specification

#### 3.2.1 Model 1

After the “4 + 7” pilot program for volume-based drug procurement in 2018 and the expansion of the alliance provinces in 2019, the national drug volume-based procurement policy has been promoted nationwide, then the volume-based policy has become normalized. This study regards pharmaceutical volume-based procurement as a quasi-experiment, with listed pharmaceutical companies included in the scope of volume-based procurement as the treatment group, and listed pharmaceutical companies not included in the scope of volume-based procurement as the control group. As of 2022, the volume-based procurement policy has been implemented for 5 years and has been implemented 8 times. Traditional difference-in-difference model cannot be simply used to distinguish the experimental group from the control group. Therefore, this study selects a multi-period DID model to study the impact of volume-based procurement policy on innovation of listed pharmaceutical companies:

$${Innovation}_{it}=\alpha +\beta {Pro}_{it}+\delta X_{it}+\gamma_{i}+v_{t}+\varepsilon_{it}$$ (1)

Where ${Innovation}_{it}$ is the innovation capability of pharmaceutical company i at period t. This study selects the innovation capability of an enterprise from two aspects: innovation input and innovation output, as defined below. ${Pro}_{it}$ is a binary variable that indicates whether pharmaceutical company i is within the scope of volume-based procurement in year t. If so, the value is 1, otherwise it is 0. $X_{it}$ is the control variable vecto. $\gamma_{i}$ is the individual fixed effect, $v_{t}$ is a time fixed effect, and $\varepsilon_{it}$ is random error item.

#### 3.2.2 Model 2

The impact of the national volume-based procurement policy on the innovation capabilities of listed pharmaceutical companies is mainly achieved through its impact on the company’s financial performance, where factors such as profitability, growth ability, operational efficiency, and solvency play an important role in the policy’s impact. This study selects return on equity (Roe), growth rate of operating revenue (GRev), total asset turnover (Ta_Turn), and asset-liability ratio (Lev) to represent the profitability, growth ability, operational efficiency, and solvency of companies, respectively. The mechanism test model is as follows:

$$Y_{it}=\alpha +\beta {\mathrm{Pr}o}_{it}+\delta X_{it}+\gamma_{i}+v_{t}+\varepsilon_{it}$$ (2)

Where $Y_{it}$ represents the profitability, growth ability, operational efficiency, and solvency of listed pharmaceutical company i. $X_{it}$ is the control variable vector.

### 3.3 Variables

#### 3.3.1 Explained variable

This study measures the innovation of companies from two aspects: innovation input and innovation output. Among them, this study selects the proportion of R&D investment in operating revenue to measure the innovation input intensity (Shefer and Frenkel, 2005; Li and Yu, 2015; Guo, 2018), denoted as RD. And this study measures innovation output from two aspects: output quantity and output quality. The natural logarithm of the number of invention patent applications plus one is selected to represents the quantity of innovation outputs (Li and Zheng, 2016; Yu et al., 2016), denoted as Pat_Quantity. The knowledge width of invention patents proposed by Zhang and Zheng (2018) is selected to represent the quality of innovation output, denoted as Pat_ Quality. The method for the knowledge width of invention patents is as follows:

$${\text{Pat}\_\text{quality}}_{i,t}=1-\sum \alpha^{2}$$ (3)

This study uses the information of the IPC classification number of invention patents to measure the quality of invention patents. IPC classification numbers are generally represented by “Section-Class-Subclass-Group-Subgroup”, such as “A00B01/00”, where A represents the section, A00 represents the class, A00B represents the subclass, A00B01 represents the group, and A00B01/00 represents the subgroup. Zhang and Zheng (2018) proposed that using the number of IPC classification numbers cannot accurately distinguish internal differences between classification numbers, which can lead to errors in the calculation of patent quality. Assuming that the IPC classification numbers of a certain patent are: “A00B01/00”, “A00B01/01”, “A00B01/02”, while the IPC classification numbers of another patent are: “A01B02/00”, “A02B03/01”, “B00C01/00”, although the number of classification numbers of these two patents is the same, they contain different information about the patent group. The former only contains information about the A00B01 group, while the latter includes information about the A01B02, A02B03, and B00C01 three groups, latter contains a wider range of patent knowledge than the former. Where α represents the proportion of each group classification in the patent classification number. The larger the ${\text{Pat}\_\text{quality}}_{i,t}$ , the wider the knowledge of the patent, then the higher the quality of the patents may be.

#### 3.3.2 Explanatory variable

The volume-based procurement policy has been officially implemented since 2018. In 2018, there were “4 + 7” pilot batch, 2019 was the expansion batch of the alliance provinces, 2020 was the second to third batch, 2021 was the fourth to sixth batch, and 2022 was the seventh batch. This study sets the national volume-based procurement policy as a binary variable, ${Pro}_{it}={Treat}_{i}\times {Post}_{t}$ . Among them, if the drugs produced by pharmaceutical company i are within the scope of the volume-based procurement, the value of ${Treat}_{i}$ is 1, if he drugs produced by pharmaceutical company i are not within the scope of the volume-based procurement, then the value of ${Treat}_{i}$ is 0. When the drugs produced by the pharmaceutical company are in the volume-based procurement batch of year t, then the value of ${Post}_{t}$ is 1 in year t and subsequent years, otherwise it is 0.

#### 3.3.3 Control variable

This study selects the following seven variables as control variables (Li and Yu, 2015; Yu and Zhong, 2019): 1) Company size (Size); 2) The return on total assets (Roa); 3) Asset liability ratio (Lev); 4) The growth rate of operating revenue (GRev); 5) Government subsidies (Subsidy); 6) The concentration of equity (Concent); 7) Turnover rate of total assets (Ta_turn).

Table 1 shows the information of the main variables.

**TABLE 1:** Main variables information.

Symbol	Name	Definition
RD	Innovation Input	R&D investment/Operating income
Pat_Quantity	Innovation Output Quantity	The natural logarithm of the number of invention patent applications plus 1
Pat_Quality	Innovation Output Quality	Calculated based on the definition of patent knowledge width in Eq. 2 by Zhang and Zheng (2018)
Pro	The volume-based procurement policy	Pro=Treatment×Pos , when the pharmaceutical firm is within the scope of volume-based procurement, the value of treatment is 1, otherwise it is 0; When the drugs produced by the pharmaceutical company are in the volume-based procurement batch of year t, the value of Post is 1 in year t and subsequent years, otherwise it is 0
Size	Company size	The natural logarithm of the total assets
Roa	Return on total assets	Net income/total assets
Grev	Growth rate of revenue	(Current year’s revenue/previous year’s revenue)-1
Ta_turn	Turnover rate of total assets	revenue/total assets
Lev	Asset liability ratio	Total liabilities/total assets
Subsidy	Government subsidy	Government subsidies received that year
Concent	Ownership concentration	The largest shareholder’s shareholding/top ten shareholders’ shareholding

Table 2 shows the data descriptive statistics of the main variables.

**TABLE 2:** Data descriptive statistics.

	Obs	Mean	Std. Dev	Min	Median	Max
*RD*	1,312	6.155	5.591	0.008	4.695	48.470
*Pat_quantity*	1,312	14.261	29.799	0.000	6.000	525.000
*Pat_quality*	1,312	0.411	0.282	0.000	0.500	0.900
*Pro*	1,312	0.132	0.338	0.000	0.000	1.000
*Ta*	1,312	79.161	146.788	1.228	36.057	1981.349
*Roa*	1,312	6.989	9.372	−71.268	6.583	128.476
*Roe*	1,312	9.119	27.303	−488.984	10.446	460.738
*GRev*	1,312	16.462	43.712	−82.497	12.688	997.801
*Ta_Turn*	1,312	0.607	0.317	0.069	0.551	2.632
*Lev*	1,312	33.461	17.021	2.929	31.343	111.816
*Subsidy*	1,312	0.354	0.549	0.000	0.160	5.507
*Concent*	1,312	54.454	17.548	14.881	53.892	97.652

## 4 Empirical results

### 4.1 Benchmark test

Table 3 shows the estimated results of the Eq. 1. The regression coefficients of *Pro* in column 1) and 2) are 2.400 and 1.690, both significant at the 1% significance level, indicating that the policy can significantly promote the R&D intensity of pharmaceutical companies. From the results in columns 3) and 4), the regression coefficients of Pro are −0.147 and −0.269, significant at the 5% and the 1% significance level, indicating that the policy significantly reduces the innovation output quantity of listed pharmaceutical companies. The results in columns 5) and 6) show that the volume-based drug procurement policy significantly improves the innovation output quality of listed pharmaceutical companies.

**TABLE 3:** Benchmark regression results.

	Innovation input		Innovation output			
			Quantity		Quality	
	(1)	(2)	(3)	(4)	(5)	(6)
*Pro*	1.182**	1.112**	−0.167**	−0.175**	0.0540**	0.0566**
	(0.525)	(0.463)	(0.0825)	(0.0837)	(0.0268)	(0.0278)
*Constant*	5.999***	6.548***	1.914***	1.646***	0.404***	0.191*
	(0.0692)	(1.858)	(0.0225)	(0.187)	(0.00761)	(0.0977)
Control variables	NO	Yes	No	Yes	No	Yes
Company dummies	Yes	Yes	Yes	Yes	Yes	Yes
Year dummies	Yes	Yes	Yes	Yes	Yes	Yes
Observations	1,312	1,312	1,312	1,312	1,312	1,312
R-squared	0.725	0.759	0.733	0.734	0.398	0.402

Note: Robust standard errors clustered at the company level in parentheses, ***p < 0.01, **p < 0.05, *p < 0.1.

### 4.2 Robustness test

#### 4.2.1 Parallel trend test

**FIGURE 1:**
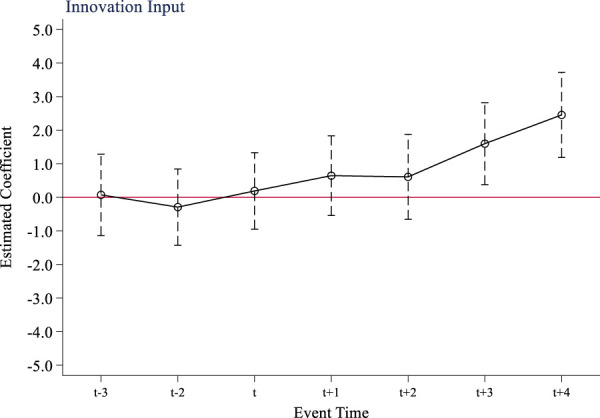
Parallel trend test of innovation input.

**FIGURE 2:**
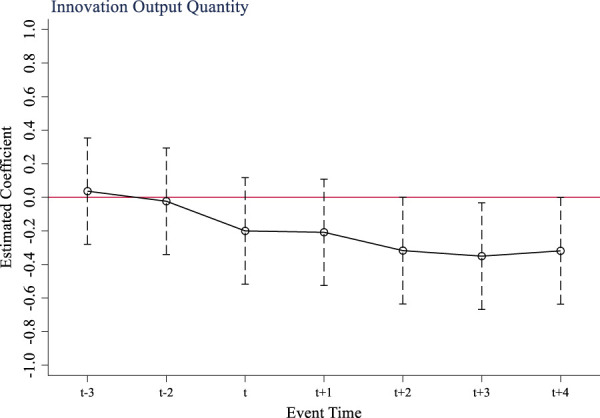
Parallel trend test of innovation output quantity.

**FIGURE 3:**
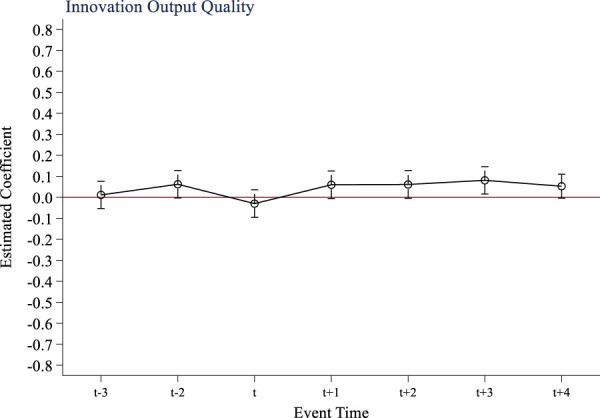
Parallel trend test of innovation output quality.

The multi-period difference-in-difference method is a quasi-experimental method that relies on parallel trend assumptions to test the causal effect of policy. If there are significant different trends in the outcome variables of the treatment group and the control group before and after the implementation of the policy, it will lead to a violation of the parallel trend assumption, and it is impossible to accurately determine whether the factors that cause the changes in the outcome variables are fully attributed to the policy. This study takes 2017, the year before the implementation of the national volume-based drug procurement policy, as the base period (t-1). Figure 1 shows the results of the parallel trend test of the policy on innovation input of listed pharmaceutical companies. From Figure 1, it can be seen that before the implementation of the policy, the difference in R&D investment intensity between companies not included in the volume-based procurement scope and companies entering the procurement scope fluctuated around 0. This supports the parallel trend assumption that there was no distinction between the treatment group and the control group prior to the policy’s implementation. After the implementation of the volume-based procurement policy, the R&D investment intensity of pharmaceutical companies in treatment group has significantly increased, indicating that the policy implementation has a stimulating effect on innovation input. Similarly, Figures 2, 3 show that the tests for quantity and quality of innovation output pass parallel trend tests as well.

#### 4.2.2 Placebo test

**FIGURE 4:**
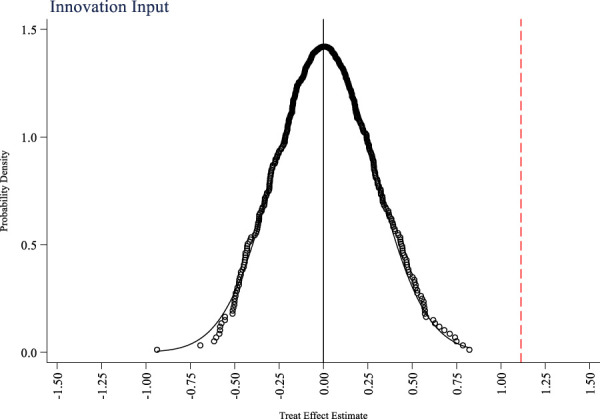
Placebo test of innovation input.

**FIGURE 5:**
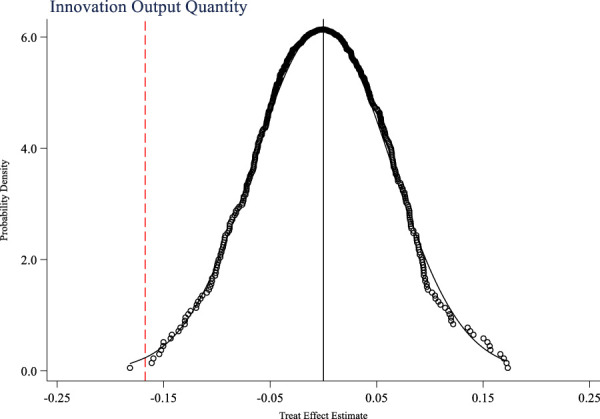
Placebo test of innovation output quantity.

**FIGURE 6:**
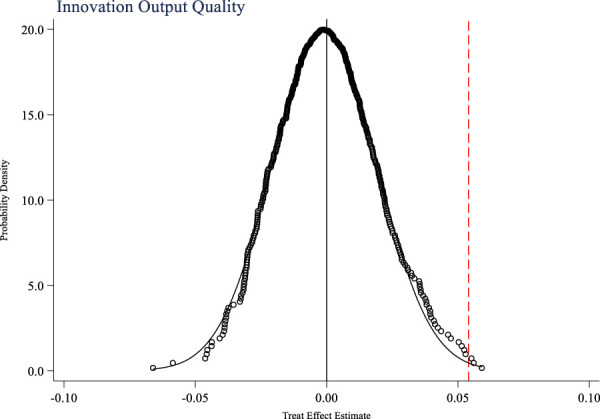
Placebo test of innovation output quality.

In addition to the national volume-based drug procurement policy, are there any other events that occur simultaneously and affect the innovation ability of pharmaceutical companies? This study takes the innovation input and output of pharmaceutical companies as research variables, and uses random sampling method to randomly set up a policy experimental group to test a placebo event that is not an actual policy as a hypothetical policy. Figures 4–6 respectively show the placebo test results for innovation input, innovation output quantity, and innovation output quality. From the figures, it can be seen that the mean of the estimated coefficient values after 500 random sampling falls around 0, indicating that the impacts of the policy on the innovation input, innovation output quantity, and innovation output quality of pharmaceutical companies are not affected by other random factors.

#### 4.2.3 PSM-DID test

**FIGURE 7:**
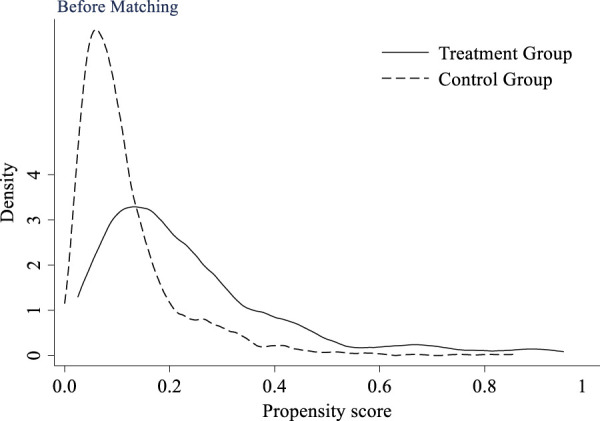
Propensity score value before matching.

**FIGURE 8:**
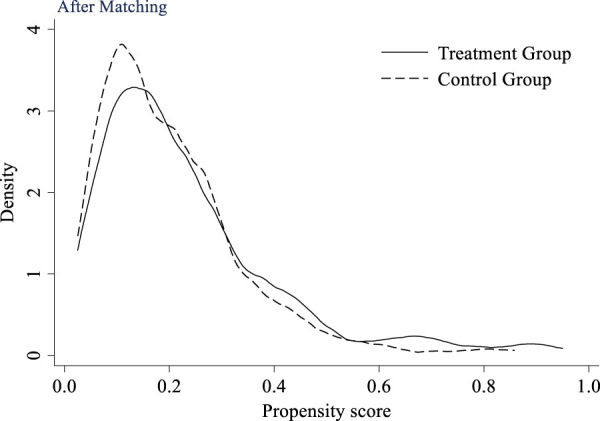
Propensity score value after matching.

Due to the fact that multi-period DID is a quasi-experimental method based on real data, the selection of research sample is difficult to achieve complete randomization, which can introduce selection bias and affect the accuracy of research conclusions. The propensity score matching method can achieve sample matching to a certain extent, reduce the impact of selection bias, and make the final research conclusion more accurate and credible. This study selects K nearest neighbor to match the treatment group, and the propensity scores before and after matching are shown in Figure 7 and Figure 8, respectively. From the figures, it can be seen that after matching, the distribution of propensity scores in the matched covariates between the control group and the treatment group is similar, and the matched treatment group and control group maintain high homogeneity in observable variables. This helps to avoid the impact of intra group heterogeneity on research results.

Table 4 shows the estimated results of PSM-DID. The results in column 1) and 2) show that the regression coefficient of the national volume-based drug procurement policy on innovation input of listed pharmaceutical companies is still significantly positive, indicating that after considering the problem of self-selection, the positive impact of the policy on innovation input is robust. The results in columns (3)–(4) and (5)–(6) also show that the results of benchmark regression are robust.

**TABLE 4:** Regression results of PSM-DID.

	Innovation input		Innovation output			
			Quantity		Quality	
	(1)	(2)	(3)	(4)	(5)	(6)
*Pro*	0.951**	0.995**	−0.160*	−0.167**	0.0541**	0.0556**
	(0.430)	(0.405)	(0.0842)	(0.0849)	(0.0272)	(0.0280)
*Constant*	6.018***	5.994***	1.936***	1.632***	0.405***	0.199*
	(0.101)	(1.534)	(0.0233)	(0.194)	(0.00784)	(0.106)
Covariates	Yes	Yes	Yes	Yes	Yes	Yes
Control variables	No	Yes	No	Yes	No	Yes
Company dummies	Yes	Yes	Yes	Yes	Yes	Yes
Year dummies	Yes	Yes	Yes	Yes	Yes	Yes
Observations	1,245	1,245	1,245	1,245	1,245	1,245
R-squared	0.748	0.779	0.733	0.735	0.405	0.410

Note: Robust standard errors clustered at the company level in parentheses, ***p < 0.01, **p < 0.05, *p < 0.1.

#### 4.2.4 Stacked DID

In traditional DID research designs, there is usually only one processing time point difference between the treatment group and the control group, but in multi-period DID, there are differences within the treatment group in the timing of receiving processing. Two-way fixed effect (TWFE) regression method treats individuals who have already been treated as a control group for untreated individuals in multi-period DID, this will result in individuals that have already been processed gaining negative weights, thereby introducing bias. In extreme cases, if the effect of negative weight group dominates, it may result in TWFE obtaining an estimate of the opposite sign to the true effect. A new estimation method, stacked DID, proposed by Cengiz et al., in 2019, is mainly used to solve the bias problem in multi-period DID. The main idea of stacked DID is to estimate the effects of each processing group separately, and then weighted average these estimation results to obtain the overall processing effect. Table 5 shows the average treatment effects of multi-period DID based on the method of Cengiz et al. (2019). From the results in Table 5, it can be seen that the impact of volume-based procurement policy on the innovation input, innovation output quantity and innovation output quality of pharmaceutical companies are also robust.

**TABLE 5:** Stacked DID estimated results.

	Innovation input	Innovation output	
		Quantity	Quality
	(1)	(2)	(3)
DID	1.470***	−0.234**	0.061**
	(0.484)	(0.106)	(0.026)
Constant	8.789***	1.418***	0.375***
	(0.467)	(0.089)	(0.034)
Contral variables	Yes	Yes	Yes
Company dummies	Yes	Yes	Yes
Year dummies	Yes	Yes	Yes
Observations	1,312	1,312	1,312
R-squared	0.794	0.729	0.398

Note: Robust standard errors clustered at the company level in parentheses, ***p < 0.01, **p < 0.05, *p < 0.1.

### 4.3 Mechanism test

The impact of the national volume-based drug procurement policy on the innovation ability of pharmaceutical companies is achieved by exerting survival pressure on them. Table 6 shows the impact of the policy on the profitability, growth ability, operational efficiency, and solvency of pharmaceutical companies in Eq. 3. The estimated results show that the volume-based drug procurement policy significantly reduces pharmaceutical companies’ profitability, growth ability, operational efficiency, and solvency. The volume-based procurement policy adjusts drug prices and the competitive mechanism of the drug market, which has a significant impact on the fundamentals of pharmaceutical firms. The survival pressure of pharmaceutical firms in the field of generic drugs is increasing, and they need to strengthen independent innovation and develop new drugs with independent intellectual property rights, in order to transition from generic drugs to the R&D of innovative drugs.

**TABLE 6:** Mechanism test.

	Survival pressure			
	Profitability	Growth ability	Operational efficiency	Solvency
	(1)	(2)	(3)	(4)
*Pro*	−4.200***	−13.28***	−0.0430**	3.122**
	(1.366)	(3.683)	(0.0211)	(1.538)
*Constant*	9.371	−32.50	0.932***	26.96***
	(8.496)	(32.77)	(0.102)	(6.011)
Control variables	Yes	Yes	Yes	Yes
Company dummies	Yes	Yes	Yes	Yes
Year dummies	Yes	Yes	Yes	Yes
Observations	1,312	1,312	1,312	1,312
R-squared	0.002	0.033	0.074	0.018

Note: Robust standard errors clustered at the company level in parentheses, ***p < 0.01, **p < 0.05, *p < 0.1.

### 4.4 Heterogeneity test

#### 4.4.1 Innovative drug companies and generic drug companies

For exploring the different impacts of the national volume-based drug procurement policy on generic and innovative drug companies, this study classifies sample companies into innovative drug manufacturing enterprises and generic drug manufacturing enterprises based on the classification of innovative drugs on Wind, with 23 innovative drug companies and 142 generic drug companies. Table 7 shows the impact of the national volume-based drug procurement policy on the innovation input and output of innovative and generic drug companies.

**TABLE 7:** Heterogeneity test between innovative and generic drug companies.

	Innovative drug			Generic drug		
	Innovation input	Innovation input		Innovation input	Innovation output	
		Quantity	Quality		Quantity	Quality
	(1)	(2)	(3)	(4)	(5)	(6)
*Pro*	4.325***	0.122	0.0213	0.895***	−0.290***	0.0967***
	(1.435)	(0.134)	(0.0231)	(0.303)	(0.0976)	(0.0285)
Constant	2.809	5.487***	0.530***	1.818	0.361	0.313***
	(10.40)	(1.507)	(0.0993)	(1.799)	(0.452)	(0.0555)
Control variables	Yes	Yes	Yes	Yes	Yes	Yes
Company dummies	Yes	Yes	Yes	Yes	Yes	Yes
Year dummies	Yes	Yes	Yes	Yes	Yes	Yes
Observations	184	184	184	1128	1128	1128
R-squared	0.376	0.161	0.115	0.193	0.038	0.020
Number of Company	23	23	23	141	141	141

Note: Robust standard errors clustered at the company level in parentheses, ***p < 0.01, **p < 0.05, *p < 0.1.

The national volume-based drug procurement policy can significantly increase R&D investment in China pharmaceutical companies. This policy enhances the willingness and ability of pharmaceutical companies to innovate by intervening in the distribution of the pharmaceutical industry and intensifying market competition. For innovative drug companies, the policy may be seen as a signal that the market is more inclined to reward innovation and efficient R&D activities, so these companies increase R&D investment to maintain a competitive edge. For generic drug companies, the policy forces them to shift from the traditional low R&D investment model to adapt to the needs of policy guidance and market competition.

The impact of the volume-based procurement policy on the innovation quantity of innovative drug companies is not significant, but it significantly reduces the innovation quantity of generic drug companies. Innovative drug companies themselves have strong motivation and ability in developing new products, and the policy does not have a great impact on their quantity. On the contrary, generic drug companies face greater pressure because their traditional R&D models and product lines may no longer be favored by the market. The volume-based procurement policy encourages these companies to reduce investment in inefficient R&D projects, thereby reducing the number of innovations.

The volume-based procurement policy significantly promotes the innovation quality of generic drug companies, but has an insignificant impact on innovative drug companies. Through an open and transparent bidding mechanism, the policy increases the competitive pressure in the generic drug market, forcing generic drug companies to seek innovation and quality improvement beyond price competition to maintain and enhance market competitiveness. This competitive effect encourages generic drug companies to pay more attention to the selection of R&D projects, optimization of processes, and cultivation of talent, thereby improving the quality and efficiency of R&D to a certain extent. In contrast, innovative drug companies, due to the high risk, long cycle, and large investment characteristics of their R&D projects, as well as the established R&D advantages and market positioning, are relatively less affected by the volume-based procurement policy. The R&D activities of innovative drug companies are more influenced by factors such as breakthroughs in basic research, technological progress, and intellectual property protection, which have a smaller direct correlation with the procurement policy.

#### 4.4.2 Bid-winner and non-winner

The national volume-based drug procurement policy has different impacts on whether pharmaceutical companies win the bid, where here winner refers to the company that wins the bid in volume-based procurement, while the non-winner refers to the company that is not included in the scope of volume-based procurement or participated in volume-based procurement but did not win the bid. For winning pharmaceutical companies, their products allow entry into the vast hospital market, ensuring high production and high revenue. However, non-winner pharmaceutical companies will face huge revenue losses and threats to survival due to being cut off from hospital market share. Table 8 shows the impact of the national volume-based drug procurement policy on the innovation input and output of bid-winner and non-winner pharmaceutical companies.

**TABLE 8:** Heterogeneity test between bid-winner and non-winner.

	Bid-winner			Non-winner		
	Innovation input	Innovation input		Innovation input	Innovation output	
		Quantity	Quality		Quantity	Quality
	(1)	(2)	(3)	(4)	(5)	(6)
*Pro*	1.236	0.406***	0.051	1.697***	−0.169**	0.061***
	(0.790)	(0.115)	(0.047)	(0.549)	(0.086)	(0.020)
Constant	−5.300	2.279***	0.467***	7.094***	1.840***	0.448***
	(11.090)	(1.327)	(0.028)	(0.805)	(0.255)	(0.014)
Control variables	Yes	Yes	Yes	Yes	Yes	Yes
Company dummies	Yes	Yes	Yes	Yes	Yes	Yes
Year dummies	Yes	Yes	Yes	Yes	Yes	Yes
Observations	480	480	480	832	832	832
R-squared	0.296	0.122	0.020	0.165	0.032	0.011
Number of Company	60	60	60	104	104	104

Note: Robust standard errors clustered at the company level in parentheses, ***p < 0.01, **p < 0.05, *p < 0.1.

The national volume-based drug procurement policy has no impact on the R&D intensity of winning companies, but has significantly positive impact on the R&D intensity of non-winning companies. For non-winning companies, the failure of key products to enter into the procurement catalog poses a significant business crisis. For the long-term development, non-winning companies can only choose to increase their efforts in innovative drug research and development, and open up the market by developing competitive innovative drugs. Non-winning companies actively adjust their strategies and invest heavily in innovative R&D, which is an inevitable choice for enterprise transformation.

The procurement policy significantly increases the number of invention patent applications of bid-winning companies, but significantly has negative impact on non-winning companies. And the procurement policy significantly improves the innovation output quality of non-winning pharmaceutical companies, but the positive impact on winning pharmaceutical companies is not significant. Non-winning companies face a cliff threat to their past business operations and product routes due to their main products not enter into the national procurement. For the long-term development, non-winning companies must adjust their innovation strategies and focus their limited resources more on developing truly competitive innovative drugs. This will inevitably lead to a shift in its innovation strategy towards a strategy of preferring quality over quantity. In contrast, the winning pharmaceutical company, due to its guaranteed revenue and relatively low survival pressure, still adopts the original innovative strategy of preferring quantity over quality.

## 5 Conclusion and recommendations

This study takes the national volume-based drug procurement policy as the entry point, selects the pharmaceutical industry in China as the research object, and uses the multi-period DID model to study the impact of the national volume-based drug procurement policy on the innovation ability of listed pharmaceutical companies. The empirical results show that the drug procurement policy has promoted the innovation input of pharmaceutical companies, reduced the innovation output quantity of pharmaceutical companies, and improved the innovation output quality of pharmaceutical companies. Looking at the specific impact mechanisms, the policy has reduced the profitability, growth capacity, operational capacity, and debt-paying ability of pharmaceutical companies, indicating that the volume-based procurement policy has forced the pharmaceutical industry to innovate by exerting survival pressure on pharmaceutical companies.

From the perspective of innovative drug companies and generic drug companies, innovative drug companies, due to the high risk, long cycle, and large investment characteristics of their R&D projects, as well as the R&D advantages and market positioning that have been established, are relatively less affected by the volume-based procurement policy. For generic drug companies, they need to adjust their strategies in the short term, pay more attention to cost control and market positioning to adapt to market and policy changes, and allocate limited resources to the R&D investment of high-quality generic drugs or innovative drugs to maintain market competitiveness. That is, the procurement has significantly promoted the innovation input of generic drug companies, reduced the output quantity of generic drug companies, and increased the quality of innovation outputs.

From the perspective of wining and non-winning companies, they face different situations and pressures, and make strategic adjustments in their respective environments, resulting in different innovation strategies. For winners, the volume-based policy has a significant promoting effect on R&D investment, but has no significant impact on the quantity and quality of innovation output. For non-winners, due to the loss of the main market share of the products, the company’s revenue has decreased, and the available R&D funds have decreased, thus placing greater emphasis on the quality of innovation output. Overall, under the national volume-based drug procurement, the winning companies still continue their original innovation strategy, while non-winning companies shift to an innovation strategy of prioritizing quality over quantity under the survival pressure.

Based on the research results in this study, there are two policy implications: 1) The national volume-based drug procurement policy should continue to be deepened and its coverage expanded. This measure is conducive to further reducing drug prices and alleviating the medical burden on patients. And it is necessary to strengthen the supervision of the entire centralized procurement process to ensure the quality and safety standards of the selected drugs, and to protect the health rights and interests of the public. 2) For innovative drugs and high-end generic drugs, it is recommended to use volume-based procurement policies with caution. This is because the research and development of innovative drugs requires a large amount of capital investment and a long period of market exclusivity to recover costs. Therefore, it is appropriate to provide these drugs with more market space and profit returns to encourage pharmaceutical companies to continue R&D and innovation, and to promote the industry towards a higher level of technological content.

Further research can be conducted in this study as follows. Firstly, this study only selects companies listed on the mainland Chinese stock market as samples, which has its limitations. Some pharmaceutical companies in China are listed on the Hong Kong and U.S. markets, and some of their pharmaceutical products also participate in volume-based procurement. In the future, these companies could be included in the sample for research. Secondly, this study only studies the volume-based procurement of generic chemical drugs and does not cover the procurement of traditional Chinese medicine or medical devices. In the future, the scope of research on volume-based procurement varieties can be expanded. Finally, the procurement of medicine is still ongoing, and this study only includes the first 7 batches of centralized procurement. It is worth considering whether there will be different conclusions as the range of centralized procurement varieties expands in subsequent procurements.

## Data Availability

The original contributions presented in the study are included in the article/supplementary material, further inquiries can be directed to the corresponding author.
